# Determinants of climate anxiety and willingness for climate-friendly behavioral change in German students: a quantitative study

**DOI:** 10.1186/s40359-026-04438-0

**Published:** 2026-04-01

**Authors:** Timothy Mc Call, Gloria Düllberg, Michel Rinderhagen, Jonas Rickermann, Laura Niediek, Kristin Liebner, Maria Leidinger, Carlotta Louisa Westfeld, Jan Kössendrup, Anna Lisa Schmid

**Affiliations:** 1 Medical School OWL, Sustainable Environmental Health Sciences, Bielefeld University, Bielefeld, Germany; 2 School of Public Health, Bielefeld University, Bielefeld, Germany

**Keywords:** Climate anxiety, Climate-friendly behavior, Climate-specific health literacy, Students, Germany

## Abstract

**Background:**

Young people are increasingly concerned about climate change, which can negatively affect their mental health. Course content about the health consequences of climate change, as taught to students of health-related subjects, is associated with increased climate anxiety. Climate anxiety can be both an understandable reaction to a real crisis that leads to climate-friendly behavior and a functional impairment that undermines the ability to act. This study aims to identify risk and protective factors for functional impairment of students due to climate anxiety, as well as factors that promote students’ willingness for climate-friendly behavioral change.

**Methods:**

Students of health-related subjects were surveyed using a quantitative online questionnaire. Age, gender, trait anxiety, personality, nature connectedness, climate-specific health literacy, and climate anxiety were recorded and analyzed using multiple linear regression analyses.

**Results:**

Students’ climate anxiety was positively and significantly associated with neuroticism, trait anxiety, connectedness to nature, female gender, knowledge of the consequences of climate change, perceived relevance of climate change, and the belief that climate change poses a personal health risk. Climate anxiety, openness, and perceived action efficacy were significantly and positively associated with students’ willingness for climate-friendly behavioral change. Adding a quadratic term for climate anxiety indicated that the relationship between climate anxiety and willingness may not be linear. Rather, willingness initially increased and then decreased slightly with rising climate anxiety.

**Conclusions:**

As our results do not indicate any protective factors against impairment due to climate anxiety, further research on this topic is advised. Future studies should give special consideration to young female students, with pronounced anxiety, neuroticism, and nature connectedness, as a potential risk group. The nature and direction of the relationship between climate anxiety and the willingness for climate-friendly behavioral change should be investigated further to determine the extent to which climate anxiety motivates or paralyzes more precisely.

**Supplementary Information:**

The online version contains supplementary material available at 10.1186/s40359-026-04438-0.

## Introduction

As the negative consequences of climate change become more visible, the number of people reporting mental health problems related to climate change has increased worldwide [1]. Young people, in particular, are concerned about climate change [2], as expressed in initiatives such as Fridays for Future. Courses for students studying health-related subjects often include information about climate change and its health consequences, which can be associated with higher levels of climate anxiety [3]. It is important not to pathologize climate-related concerns, as climate change is a real threat [4]. Climate-related fears can increase the willingness for climate-friendly behavioral change in order to address the perceived danger [5]. However, climate anxiety can be associated with functional impairment [6, 7] and thus undermine one’s own ability to act. Understanding the risk factors for these negative effects is essential for developing targeted interventions. It is also essential to identify protective factors against climate anxiety-related impairment to strengthen the resilience of vulnerable groups. According to Reismann et al. [8], one potentially protective new construct is climate-specific health literacy. With this study we aim to determine which factors can be used to predict (1) climate anxiety-related impairment and (2) the willingness for climate-friendly behavioral change, particularly among German students studying health-related subjects. To this end, we want to identify risk and protective factors for climate anxiety, and determine the importance of climate anxiety and other factors in promoting climate-friendly behavior. Among other measures, we will test the applicability of a new questionnaire to assess climate-specific health literacy [9].

## Background

### Climate anxiety among students

#### Climate anxiety

The term eco-anxiety is used to describe the anxiety caused by ecological crises. A specific form of eco-anxiety related to anthropogenic climate change is climate anxiety [10]. Because climate change is a real threat and it is reasonable to be concerned [4], anxiety about climate change is not necessarily pathological [11]. Clayton and Karazsia [6] developed the Climate Anxiety Scale (CAS) to assess climate anxiety, which is associated with cognitive-emotional and functional impairment. According to a meta-analysis, this type of impairment is negatively correlated with mental well-being [12]. Among German students, links between climate-associated worries and mental health problems, such as depressive symptoms, sleep difficulties, and self-reported somatic symptoms have also been shown [13]. Similarly, German medical students also experience considerable stress related to climate change. Although this was not (yet) reflected in depressive, anxious, or traumatic symptoms, it was negatively related to resilience factors that prevent mental disorders [14]. These findings emphasize the relevance of addressing climate anxiety in the face of advancing climate change. Other emotional reactions to climate change besides anxiety include anger, guilt, and contempt (see for example the Inventory of Climate Emotions (ICE) by Marczak et al. [15]).

#### Students of health-related subjects as risk group for climate anxiety?

The current state of research suggests that young people, in particular, are affected by negative emotions, specifically anxiety, in connection with climate change. An international study by Hickman et al. [2] shows that 59% of participating adolescents and young adults aged 16 to 25 from ten different countries are very or extremely worried about climate change. These feelings about climate change negatively affected the daily lives and functioning of 45% of respondents. In addition, international studies have concluded that there is a significant negative correlation between climate anxiety and age [16–19], meaning a younger people tend to experience higher levels of climate anxiety. Two German studies confirm this negative correlation between climate anxiety and age [20, 21], while another German study found no correlation between age and climate anxiety [7].

Evidence suggests that climate concerns may not only be associated with young age, but also with student status. According to a Portuguese study, students report moderate to high levels of environmental worry [22]. A study by Söder et al. [13] additionally showed that moderate worry about climate change is prevalent among German students: on a scale of one (no worry) to five (great worry), the average level of worry about climate change was 2.8. Among students of medicine and health sciences, the mean score was higher, at 2.9 [13]. This could indicate that students of health-related subjects are disproportionately affected by climate change worry. Consistent with this assumption, a study of Turkish nursing students found that the mean score for climate change worry was 3.1 on the same scale [23]. 27% of the participants in this study had already taken an environmental health course [23]. Courses covering health-related subjects often include information about climate change and its health consequences. According to a Mexican study, this knowledge is associated with higher levels of climate anxiety among students [3]. Similarly, a study of UK students found rather low overall levels of climate anxiety while students of environmental degrees reported higher levels of climate anxiety [24]. Therefore, acquiring climate-specific health literacy through health-related or environmental studies appears to be a potential risk factor for heightened climate anxiety among students.

### Factors associated with climate anxiety

#### Gender and climate anxiety

To identify potential risk groups for negative consequences of climate anxiety, it is important to know what factors are associated with these consequences. As mentioned above, these factors include being young, but also being female: Several studies have shown that climate anxiety is more prevalent among women than men [7, 17, 18], including university students [3].

#### Personality traits and climate anxiety

The level of climate anxiety may depend on students’ personality traits. According to a meta-analysis by Cipriani et al. [25], concern about climate change is positively correlated with openness and neuroticism. A study by Tucholska et al. [26] found a significant positive correlation between climate anxiety and neuroticism, as well as between climate anxiety and openness, in adults from Poland. While neuroticism was identified as a significant positive predictor of cognitive-emotional impairment because of climate anxiety, conscientiousness was identified as a significant negative predictor of functional impairment because of climate anxiety [26].

#### Trait Anxiety and climate anxiety

In addition to the aforementioned personality traits, studies suggest that trait anxiety may be associated with the level of climate anxiety in students. Numerous studies suggest a significant positive correlation between functionally impairing climate anxiety and general anxiety or anxiety disorder symptoms [7, 16, 19, 27, 28], including among US students [29] and among a young, educated sample from Germany [30]. Another German study showed that the extent of functionally impairing climate anxiety is associated with general anxiety and depression, as well as a depression and/or anxiety disorder diagnosis [31].

#### Nature connectedness and climate anxiety

Another potential factor influencing the level of climate anxiety among students is their connection to nature. In a study by Curll et al. [32], a significant positive correlation was found between connectedness to nature and worry about climate change among Australian adults surveyed after a severe bushfire season. This finding was supported by Canadian and UK studies that found significant positive correlations between climate anxiety and connectedness to nature [28] and nature relatedness [19] in adults. These international research findings were confirmed by a German study that identified connectedness to nature as a possible risk factor for impairment due to climate anxiety [33].

#### Climate-specific health literacy and climate anxiety

As mentioned earlier, acquiring knowledge about the health consequences of climate change can affect students’ climate anxiety. This relates to the concepts of environmental and climate-specific health literacy. Environmental literacy is defined as the process that forms or strengthens an individual’s environmental values and competencies in problem-solving for environmental issues [34]. Climate-specific health literacy, on the other hand, is characterized by knowledge about the health risks of climate change and the health co-benefits of climate-friendly behavior. It also involves the emotional integration of knowledge and feelings of concern about climate change and health, as well as the ability to translate this knowledge into climate-friendly actions [8]. In an exploratory study, Albrecht et al. [9] attempted to capture climate-specific health literacy using a self-constructed questionnaire, which in addition to the elements proposed by Reismann et al. [8], also included the barriers and enabling factors of climate-specific health literacy. To our knowledge, the relationship between climate anxiety and climate-specific health literacy among students has not yet been investigated. However, there are indications in the literature that suggest that this correlation should be analyzed.

While studies with adults from the general population have shown a negative correlation between climate anxiety and climate knowledge [16, 28], a study with Mexican university students found that knowledge about climate change was significantly positively related to climate anxiety scores [3]. A quasi-experimental pretest-posttest study of nursing students in Istanbul that was conducted to investigate the effects of a course containing information about the causes and consequences of climate change and proposals to mitigate it, showed similar results. After taking the course, students demonstrated a statistically significant increase in knowledge and attitudes towards global warming and notably, also an increase in eco-anxiety [35].

### Factors associated with the willingness for climate-friendly behavioral change

#### Gender, age, and willingness to change behavior

In addition to climate anxiety levels, the aforementioned characteristics can also be associated with the willingness for climate-friendly behavioral change. With regard to demographic factors, current studies indicate that pro-environmental behavior is significantly positively associated with gender [1] and age [19], meaning that being female and older is associated with more pro-environmental behavior. However, it is important to note that willingness to engage in environmentally friendly behavior does not always go hand in hand with actual behavior [36].

#### Personality traits and willingness to change behavior

Personality traits could also be associated with students’ willingness to change climate-friendly behavior. A meta-analysis by Cipriani et al. [25] shows a positive correlation between proactivity toward climate change and openness, extraversion, agreeableness, and neuroticism. A Polish study by Tucholska et al. [26], demonstrated a significant positive association between openness, extraversion, and conscientiousness and pro-environmental activity, while neuroticism and openness were significantly associated with planned pro-environmental activity [26].

In a British birth cohort study, longitudinal data were analyzed to identify associations between personality traits and pro-environmental behaviors. Strong positive associations were found between agreeableness, openness and the total number of pro-environmental actions taken. A weaker positive association was found between extraversion and the total number of pro-environmental actions taken, and little to no association was found between conscientiousness and the total number of pro-environmental behaviors taken [37]. A meta-analysis by Soutter et al. [38] shows that openness, conscientiousness, agreeableness, and extraversion significantly correlate with pro-environmental behavior. All in all, all big five personality traits have been linked to climate-friendly or pro-environmental behavior.

#### Climate anxiety and willingness to change behavior

Climate anxiety can be understood as an adaptive reaction to a real threat accompanied by behaviors that mitigate the danger [5, 39]. Therefore, for some people, climate anxiety can act as a driver to take climate action [40]. Numerous studies have found a significant positive correlation between climate anxiety and pro-environmental behavior in adults [1, 17–19, 26, 28, 41, 42]. Current studies with German adults confirm this association [7, 30, 43].

#### Climate-specific health literacy and willingness to change behavior

In addition to anxiety, knowledge and competence in the field of climate change and health can also lead to meaningful responses to protect the climate. For example, a study of physicians and nurses from Germany found that participants who rated their knowledge of the consequences of climate change higher were more willing to eat a vegetarian diet or volunteer for sustainability than participants who rated their knowledge lower [9]. This trend was not only observed among medical practitioners, but also among patients: In a study by Reismann et al. [8], the majority of participating patients stated that they would be willing to adopt climate-friendly behavior if their physician informed them about the health risks associated with climate change. This connection was also evident among students: German students who were well informed about the health consequences of climate change were more willing to adopt climate-friendly behavior. This willingness manifested itself in actual behavior, like choosing environmentally friendly means of transport or adopting a sustainable diet offering health co-benefits [44].

### Problem definition and aims

To our knowledge, research into climate anxiety and its associated cognitive-emotional and functional impairments, as well as the factors influencing the extent of these impairments, is limited among German students, especially those studying health-related subjects. Given possible risk factors such as a young age [20, 21] and high climate knowledge [3], it is important to determine the extent to which they are affected by climate anxiety impairment. Students may need support in dealing with their climate-related emotions. In addition, in order to specifically prevent negative effects of climate anxiety, it should be determined which factors leave students vulnerable to climate anxiety-related impairment and which factors offer protection.

The study therefore aims to identify potential risk and protective factors for the cognitive-emotional and functional impairments due to climate anxiety in this target group. To this end, we will analyze the relevance of personality traits, trait anxiety, connectedness to nature, and parts of climate-specific health literacy for the impairment caused by climate anxiety. The second aim of the study is to examine the factors associated with German students’ willingness to change their behavior toward more climate-friendly practices, to identify potential strategies to promote such behavioral changes in this group. To this end, we will examine the association of climate anxiety, personality traits, and the belief that one’s own behavior has an impact on climate change with the extent to which students are willing to change their behavior. These aims translate into the following two research questions:

1. How are personality traits, trait anxiety, connectedness to nature, and climate-specific health literacy associated with climate anxiety?
2. How are climate anxiety, personality traits, and the belief that one’s own behavior has an impact on climate change associated with the willingness for climate-friendly behavioral change?

## Methods

### Participants

The study was open to all students aged 18 or over who were attending a German higher education institution at the time of the invitation. To recruit students, we sent a call for participation to 572 health-related faculties at universities and other higher education institutions across Germany, who then forwarded the study invitation to their students. Based on an a priori power calculation using G*Power (Version 3.1.9.7), we identified a target sample size of 435 for the planned regression analysis with the most predictors (see Appendix). We oversampled to account for potential missing data. Of the total 528 eligible students who participated, 365 identified as female, 153 as male, and 10 as neither or diverse. The students’ ages ranged from 18 to over 50 years, but most were between 21 and 30 years old on average. Taking part in the study was voluntary. All participants consented to participate in the study after being informed of its objectives and procedures. No financial or other compensation was provided.

### Materials and procedure

Interested students followed the invitation link to access the online study in the environment and on the device of their choice. After reading the study information and providing their informed consent, participants filled out the questionnaire. The first set of questions asked participants to provide information on whether they were studying at a German higher education institution (yes/no), their age (18–20 years; 21–25 years; 26–30 years; … or 50 + years old), and their gender identity (female; male; or diverse).

A second set of questions was used to assessed the participants’ trait anxiety, personality, and nature connectedness using the German-language versions of the short form of the State-Trait-Anxiety Inventory (STAI) [45], the 10-item short version of the Big Five Inventory (BFI-10) [46], and the Connectedness to Nature Scale for Adolescents (CNS-A) [47], respectively. The STAI has ten items in which participants rate anxiety-related statements on an 8-point Likert scale ranging from 1 “almost never” to 8 “almost always” (e.g. “I get tired quickly.”). The BFI-10 has ten items in which participants rate statements on a 5-point Likert scale ranging from 1 “not at all applicable” to 5 “fully applicable”, which allow inferences about the participants’ personality across five subscales: Extraversion (e.g. “I see myself as someone who is outgoing, sociable.”), Agreeableness (e.g. “I see myself as someone who is generally trusting.”), Conscientiousness (e.g. “I see myself as someone who does a thorough job.”), Neuroticism (e.g. “I see myself as someone who gets nervous easily.”), and Openness (e.g. “I see myself as someone who has an active imagination.”). Lastly, when answering the CNS-A, participants rated 14 nature-related statements on a 7-point Likert scale ranging from 1 “strongly disagree” to 7 “strongly agree” (e.g. “I feel one with nature.”).

The third set of questions focused on the participants’ climate-specific health literacy which was assessed using the exploratory questionnaire developed by Albrecht et al. [9]. This questionnaire assesses different aspects of climate-specific health literacy. We utilized five single 5-point Likert scale items from this questionnaire addressing the participants self-rated knowledge of the consequences of climate change (knowledge; “How would you rate your level of knowledge regarding the general consequences of climate change?”), their perceived relevance of climate change (relevance; “How relevant does climate change seem to you at the moment?”), whether participants feel their actions have an impact on climate change (action efficacy; “Do you think that your own (consumption) behavior has an impact on climate change?”), and whether participants believe climate change poses a personal (personal risk; “Do you see climate change as a risk factor to your own (long-term) health (e.g. infectious diseases, skin cancer, heat stress)?”) or global health risk (global risk; “Do you consider climate change to be the cause of global health problems (e.g. air pollution, food shortages, pandemics)?”). We also used six 5-point Likert scale items to assess the participants willingness to engage in different climate-friendly behaviors such as using a bicycle instead of a car, using public transportation instead of private transportation, switching to a vegetarian diet, switching to a vegan diet, getting involved in a community or organization that promotes sustainability or environmental protection, and paying more for sustainable products.

Finally, participants answered questions about their level of climate anxiety using the German version of the CAS [7]. This scale has 13 items, with participants rating statements describing their cognitive-emotional (e.g. “Thinking about climate change makes it difficult for me to concentrate.”) and functional impairment (e.g. “My concerns about climate change undermine my ability to work to my potential.”) due to climate anxiety, on a 7-point Likert scale ranging from 1 “strongly disagree” to 7 “strongly agree”.

The STAI (α = 0.88), CNS-A (α = 0.81), and CAS (α = 0.90) scales showed good internal consistency, while the items assessing the willingness for climate-friendly behavioral change showed acceptable internal consistency (α = 0.76). In contrast, the internal consistency of the five BFI-10 subscales was mixed: Extraversion (α = 0.77), Agreeableness (α = 0.27), Conscientiousness (α = 0.47), Neuroticism (α = 0.66), and Openness (α = 0.59). Students could participate from late May to early June 2024. The questionnaire was administered exclusively online using evasys (https://evasys.de/). Preceding the data collection, the study was assessed and then approved by the Ethics Committee of Bielefeld University (Application No. 2024 − 119).

### Data analysis

Before analyzing the data, we recoded reversed items and calculated the mean scores for the personality subscales (Extraversion, Agreeableness, Conscientiousness, Neuroticism, and Openness), nature connectedness, climate anxiety, and the willingness for climate-friendly behavioral change. As per the scale instructions, we calculated the total percentage of agreement for the STAI items (0–100), with higher scores indicating higher trait anxiety. We also created dummy variables for gender (female and diverse) to use in the regression analyses. Additionally, for the Likert-type items serving as independent variables, we merged the two lowest response categories, as only a small proportion of participants selected them. This was done to ensure sufficient statistical power for their use as reference categories.

To answer our research questions, we conducted several multiple linear regression analyses (MLRA) on the data. A first MLRA indicated that none of the personality subscales were significantly associated with climate anxiety, but trait anxiety was. Additionally, trait anxiety and neuroticism had a high correlation (0.70). Therefore, we conducted two separate MLRAs to answer the first research question: How are personality traits, trait anxiety, connectedness to nature, and climate-specific health literacy associated with climate anxiety? One MLRA used trait anxiety as the independent variable, and the other used the five personality subscales as the independent variables. Both analyses also included age, gender, climate-specific health literacy (knowledge, relevance, action efficacy, personal risk, and global risk), and connectedness to nature as additional independent variables and climate anxiety as the dependent variable. Because the assumption of homoscedasticity was not met for either model, we used bootstrapping with 5000 samples for the analyses. All other assumptions (normally distributed residuals, multicollinearity, autocorrelation, and extreme outliers) were satisfied.

To answer the second research question, we conducted another MLRA, using the willingness for climate-friendly behavioral change as the dependent variable. This analysis examined the association between climate anxiety, personality traits, and the belief that one’s own behavior has an impact on climate change and the willingness for climate-friendly behavioral change. We used age, gender, climate anxiety, the personality subscales, and action efficacy as independent variables. All assumptions were met for this analysis.

Finally, we estimated the same MLRA with an additional quadratic term added for climate anxiety. This assessed a possible non-linear relationship between climate anxiety and the willingness for climate-friendly behavioral change was. This model also met all assumptions.

## Results

A total of 528 students answered the survey. Excluding those with missing values in the analytical sample resulted in an analytical sample size of *N* = 470. Table 1 provides an overview of the sample characteristics. Most of the participants identified as female (*n* = 321; 68%), while 30% identified as male (*n* = 140), and 1.9% (*n* = 9) identified as neither male nor female. The majority were aged between 21 and 25 (*n* = 254; 54%); 23% were aged between 26 and 30 years (*n* = 108); 8.7% between 18 and 20 years (*n* = 41), 6.8% were aged between 31 and 35 years (*n* = 32), 4.7% were aged between 36 and 40 years (*n* = 22), and lastly, 1.3% were older than 40 years (*n* = 13).

Regarding their knowledge of climate change consequences, more than 70% of the participants rated their knowledge as high (*n* = 242; 51%) or very high (*n* = 97; 21%). 21% rated their knowledge as medium (*n* = 99), and less than 10% rated it as low or very low (*n* = 32; 6.8%). Nearly 90% viewed the relevance of climate change as very urgent (*n* = 348; 73%) or urgent (*n* = 69; 15%). Conversely, fewer than 5% viewed its relevance as rather insignificant (*n* = 24; 4.5%).

Regarding their action efficacy, 77% of the participants answered that they feel their actions have an impact on climate change, with 48% answering “Yes” (n = 224), and 29% answering “Yes, very” (n = 138). 8.3% stated that they do not feel that their actions have an impact on climate change (8.3%, n = 39). 77% of the participants stated that they see climate change as a cause of global health problems (climate change as a global risk). Specifically, 34% (n = 160) answered “Yes” and 43% (n = 204) answered “Yes, very.” Meanwhile, 11% (n = 51) stated that they do not see climate change as a global health risk. When asked whether they see climate change as a risk factor for their own health, 33% answered “Yes” (n = 154) and 36% answered “Yes, very” (n = 171), while 17% did not see it as a risk factor (n = 81).

In terms of personality traits, the sample demonstrated the following mean scores for each trait: extraversion 3.12 (SD = 0.99), agreeableness 3.29 (SD = 0.81), conscientiousness 3.61 (SD = 0.80), neuroticism 3.05 (SD = 0.96), and openness 3.64 (SD = 1.00). The mean score for connectedness to nature in this sample was 5.04 (SD = 0.82), for trait anxiety it was 43 (SD = 17), and for climate anxiety, it was 2.38 (SD = 1.14). Regarding the willingness for climate friendly behavioural change, the sample had a mean score of 3.93 (SD = 0.74).

**Table 1:** Sample characteristics

Characteristic		*N* = 470^1^	Mean Score (SD): Climate Anxiety	*p*-value^2^	Mean Score (SD): Willingness for climate-friendly behavioral change	*p*-value^2^
**Age**	18–20 years	41 (8.7%)	2.35 (1.21)	0.21	3.76 (0.83)	0.567
	21–25 years	254 (54%)	2.45 (1.12)		3.94 (0.72)	
	26–30 years	108 (23%)	2.47 (1.12)		4.05 (0.67)	
	31–35 years	32 (6.8%)	1.98 (0.89)		3.83 (0.83)	
	36–40 years	22 (4.7%)	2.03 (0.75)		3.84 (0.95)	
	above 40 years	13 (2.8%)	1.93 (0.88)		3.63 (0.83)	
**Gender**	male	140 (30%)	1.93 (0.98)	0.261	3.69 (0.83)	0.36
	female	321 (68%)	2.56 (1.16)		4.02 (0.69)	
	other	9 (1.9%)	2.95 (0.88)		4.28 (0.34)	
**Knowledge of climate change consequences**	very poor - poor	32 (6.8%)	1.98 (1.09)	< 0.001	3.61 (0.85)	< 0.001
	decent	99 (21%)	2.00 (0.83)		3.64 (0.78)	
	good	242 (51%)	2.41 (1.14)		4.00 (0.68)	
	very good	97 (21%)	2.83 (1.29)		4.15 (0.72)	
**Relevance of climate change**	not at all important – less important	21 (4.5%)	1.20 (0.32)	< 0.001	2.49 (0.88)	< 0.001
	moderately important	32 (6.8%)	1.57 (0.91)		2.94 (0.64)	
	very important	69 (15%)	1.76 (0.63)		3.59 (0.67)	
	extremely important	348 (73%)	2.65 (1.15)		4.17 (0.53)	
**Action Efficacy**	no effect – weak effect	39 (8.3%)	1.60 (0.77)	< 0.001	3.11 (1.03)	< 0.001
	neither nor	69 (15%)	2.16 (0.97)		3.78 (0.74)	
	some effect	224 (48%)	2.48 (1.18)		3.97 (0.69)	
	severe effect	138 (29%)	2.55 (1.16)		4.16 (0.55)	
**Climate change as a global risk**	not at all - unlikely	51 (11%)	1.76 (1.07)	< 0.001	3.11 (1.06)	< 0.001
	neither nor	55 (12%)	2.06 (0.96)		3.72 (0.87)	
	yes, mostly	160 (34%)	2.23 (1.07)		3.91 (0.62)	
	yes, a lot	204 (43%)	2.74 (1.16)		4.20 (0.49)	
**Climate change as a personal risk**	not at all - unlikely	81 (17%)	1.54 (0.67)	< 0.001	3.35 (0.94)	< 0.001
	neither not	64 (14%)	2.32 (1.19)		3.86 (0.78)	
	yes, mostly	154 (33%)	2.31 (1.01)		3.95 (0.66)	
	yes, a lot	171 (36%)	2.87 (1.18)		4.21 (0.50)	
**Personality: Extraversion**		3.12 (0.99)		0.157		0.289
**Personality: Agreeableness**		3.29 (0.81)		0.823		0.439
**Personality: Conscientiousness**		3.61 (0.80)		0.13		0.009
**Personality: Neuroticism**		3.05 (0.96)		< 0.001		0.014
**Personality: Openness**		3.64 (1.00)		0.004		< 0.001
**Connectedness to Nature**		5.04 (0.82)		< 0.001		< 0.001
**Trait Anxiety**		43 (17)		< 0.001		< 0.001

^1^n (%); Mean (SD) ^2^ χ^2^ test; Kendall’s tau test

We conducted two MLRAs with bootstrapping to analyze the association between age, gender, climate-specific health literacy (knowledge, relevance, action efficacy, personal risk, and global risk), trait anxiety, personality (extraversion, agreeableness, conscientiousness, neuroticism, and openness), and connectedness to nature and climate anxiety (Table 2). Both the model including trait anxiety (*F*(24, 445) = 13.58, *p* < .001, *adjusted R*^*2*^ = 0.39, *AIC* = 1.254) and the model including personality (*F*(28, 441) = 9.31, *p* < .001, *adjusted R*^*2*^ = 0.33, *AIC* = 1.302) were significant and explained 39% and 33% of the variance, respectively. In both models female gender, knowledge, relevance, personal risk, connectedness to nature, and either trait anxiety or neuroticism were significantly associated with climate anxiety (Table 2). Age was only significant in the personality model. On average, participants aged 36–40 reported a 0.42 lower score (95 CI [-0.84, -0.00]) in climate anxiety than those 18–20 years old. This means that being female, perceiving oneself as very knowledgeable about the consequences of climate change, perceiving climate change as highly relevant, believing that climate change poses a threat to personal health, and feeling connected to nature were associated with increased climate anxiety among students. Similarly, students who reported being more neurotic or having higher trait anxiety, also reported having higher climate anxiety.

**Table 2:** Multiple linear regression models for the outcome of climate anxiety

Characteristic		Model 1: Trait Anxiety		Model 2: Personality	
		Beta	95% CI^1^	Beta	95% CI^1^
**Age** (*Ref.: 18–20*)	21–25 years	0.05	-0.21, 0.30	-0.02	-0.31, 0.25
	26–30 years	-0.08	-0.37, 0.20	-0.10	-0.42, 0.19
	31–35 years	-0.30	-0.63, 0.07	-0.34	-0.73, 0.03
	36–40 years	-0.37	-0.77, 0.02	-0.42	-0.84, -0.00*
	above 40 years	-0.22	-0.73, 0.64	-0.40	-0.91, 0.54
**Gender** (*Ref.: male*)	female	0.29	0.12, 0.47*	0.31	0.10, 0.51*
	diverse	0.50	-0.09, 1.11	0.58	-0.12, 1.22
**Knowledge of climate change consequences** (*Ref: very poor – poor*)	decent	-0.11	-0.41, 0.19	-0.10	-0.42, 0.19
	good	0.11	-0.18, 0.39	0.13	-0.18, 0.42
	very good	0.44	0.1, 0.78*	0.48	0.11, 0.85*
**Relevance of climate change** (*Ref: not at all important – less important*)	moderatly important	0.12	-0.27, 0.54	0.08	-0.32, 0.49
	very important	0.07	-0.35, 0.47	0.10	-0.38, 0.50
	extremely important	0.56	0.14, 0.98*	0.63	0.20, 1.05*
**Action Efficacy** (*Ref: no effect – weak effect*)	neither nor	0.07	-0.29, 0.37	0.06	-0.31, 0.40
	some effect	0.17	-0.18, 0.44	0.19	-0.15, 0.50
	severe effect	0.07	-0.30, 0.37	0.00	-0.36, 0.35
**Climate change as a global risk** (*Ref: not at all – unlikely*)	neither nor	-0.19	-0.63, 0.25	-0.14	-0.59, 0.29
	yes, mostly	-0.29	-0.71, 0.09	-0.23	-0.66, 0.14
	yes, a lot	-0.17	-0.61, 0.23	-0.10	-0.56, 0.29
**Climate change as a personal risk** (*Ref: not at all – unlikely*)	neither nor	0.47	0.16, 0.79*	0.43	0.1, 0.76*
	yes, mostly	0.26	0.01, 0.52*	0.30	0.03, 0.56*
	yes, a lot	0.50	0.23, 0.79*	0.52	0.23, 0.81*
**Trait Anxiety**		0.02	0.02, 0.03*		
**Connectedness to Nature**		0.22	0.10, 0.34*	0.21	0.07, 0.33*
**Extraversion**				-0.05	-0.15, 0.05
**Agreeableness**				0.00	-0.11, 0.11
**Conscientiousness**				0.03	-0.09, 0.14
**Neuroticism**				0.26	0.14, 0.36*
**Openness**				0.02	-0.08, 0.11
Adjusted R²		0.39		0.33	
AIC		1,254		1,302	

^1^CI = (Bootstrapped) Confidence Interval; * for significance (*p* < .05)

To analyze the association between age, gender, climate anxiety, personality (extraversion, agreeableness, conscientiousness, neuroticism, and openness), and perceived action efficacy and the willingness for climate-friendly behavioral change, we conducted another MLRA. The model used was significant (*F*(16, 453) = 15.35, *p* < .001, *adjusted R*^*2*^ = 0.33) and explained 33% of the variance. Climate anxiety (*B* = 0.27, *95% CI* [0.22, 0.33], *p* < .001), openness (*B* = 0.10, *95% CI [0.04*,* 0.15]*, *p* = .001), and higher values in perceived action efficacy (all: *p* < .001) were significantly associated with the willingness for climate-friendly behavioral changes. This means that students who felt more emotionally affected by climate anxiety, who were more open to new experiences, and who felt that their actions have an impact on climate change, were overall more willing to change their climate-friendly behavior. Additionally, those aged 26–30 reported a 0.28-point higher willingness for behavioral change on average (*95% CI*: [0.06, 0.50], *p* = .012) than those aged 18–20 (Table 3).

**Table 3:** Multiple linear regression models for the outcome of willingness for climate-friendly behavioral change

Characteristic		Model 3: Willingness for climate-friendly behavioral change			Model 4: Climate Anxiety as a quadratic term		
		Beta	95% CI^1^	*p*-value	Beta	95% CI^1^	*p*-value
**Age** (*Ref.: 18–20*)	21–25 years	0.17	-0.03, 0.38	0.093	0.16	-0.04, 0.37	0.11
	26–30 years	0.28	0.06, 0.50	0.012	0.26	0.04, 0.48	0.018
	31–35 years	0.24	-0.05, 0.52	0.11	0.22	-0.06, 0.50	0.13
	36–40 years	0.16	-0.16, 0.48	0.3	0.13	-0.18, 0.45	0.4
	above 40 years	-0.02	-0.40, 0.37	> 0.9	-0.03	-0.41, 0.34	0.9
**Gender** (*Ref.: male*)	female	0.09	-0.05, 0.22	0.2	0.07	-0.07, 0.20	0.3
	diverse	0.28	-0.15, 0.71	0.2	0.22	-0.21, 0.64	0.3
**Climate Anxiety**		0.27	0.22, 0.33	< 0.001	0.71	0.50, 0.93	< 0.001
**Climate Anxiety** ^**2**^					-0.07	-0.11, -0.04	< 0.001
**Extraversion**		0.00	-0.06, 0.06	> 0.9	0.00	-0.06, 0.06	> 0.9
**Agreeableness**		0.00	-0.07, 0.07	> 0.9	0.00	-0.07, 0.07	> 0.9
**Conscientiousness**		0.03	-0.04, 0.11	0.4	0.04	-0.03, 0.11	0.3
**Neuroticism**		-0.07	-0.13, 0.00	0.053	-0.09	-0.15, -0.02	0.011
**Openness**		0.10	0.04, 0.15	0.001	0.09	0.03, 0.15	0.002
**Action Efficacy** (*Ref: no effect – weak effect*)	neither nor	0.53	0.28, 0.77	< 0.001	0.48	0.24, 0.72	< 0.001
	some effect	0.64	0.42, 0.86	< 0.001	0.60	0.38, 0.81	< 0.001
	severe effect	0.78	0.55, 1.0	< 0.001	0.73	0.51, 0.96	< 0.001
Adjusted R²		0.33			0.35		
AIC		888			872		

^1^CI = Confidence interval

**Fig. 1:**
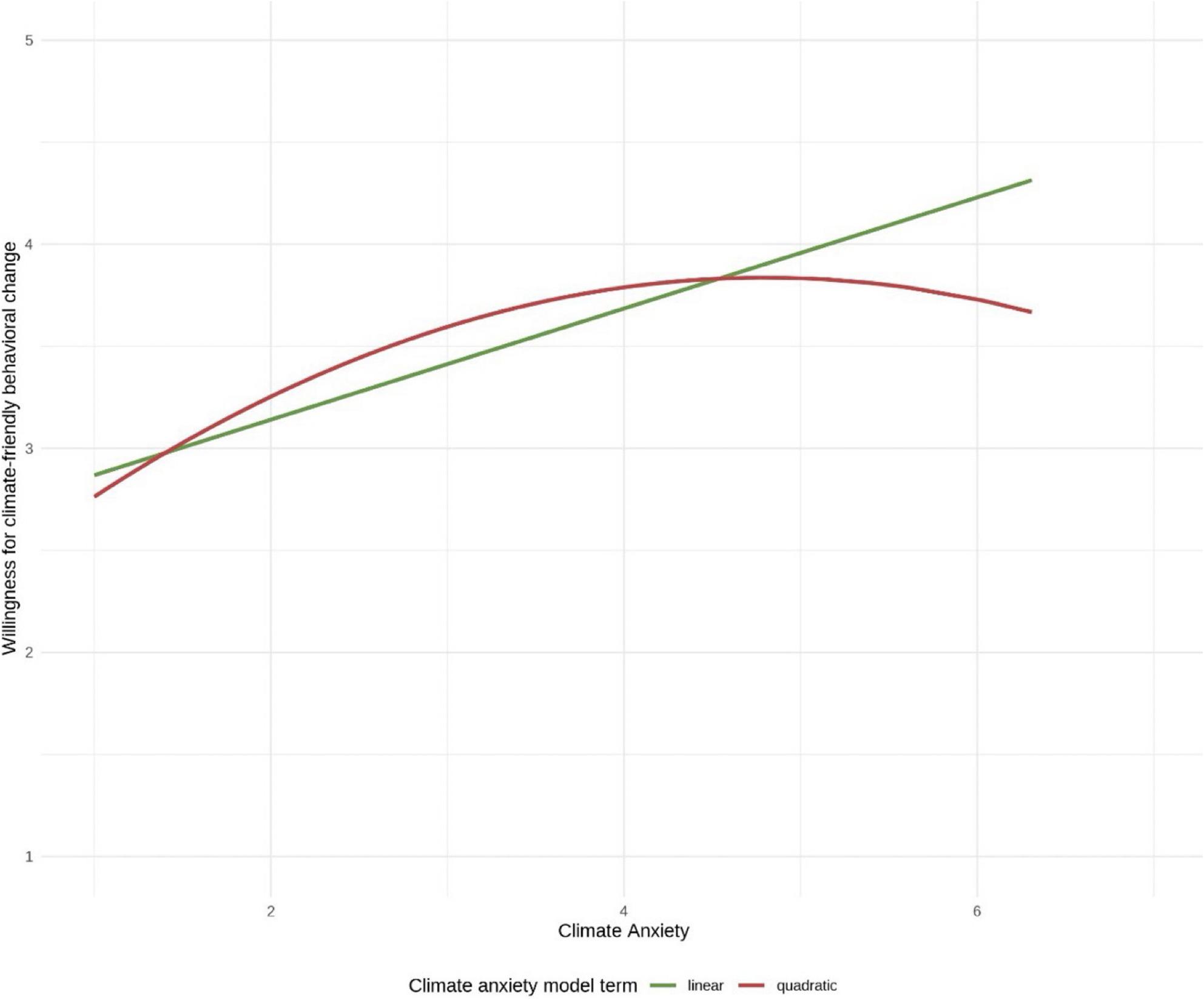
Predicted values of the linear and non-linear marginal effects of climate anxiety on the willingness for climate-friendly behavioral change

Adding a quadratic term for climate anxiety to account for a possible non-linear relationship between climate anxiety and the willingness for climate-friendly behavioral change resulted in an improved model fit (*F*(17, 452) = 16.00, *p* = < 0.001, *adjusted R*^*2*^ = 0.35, *AIC* = 872). While the coefficient for climate anxiety (*B* = 0.71, *95% CI* [0.50, 0.93], *p* = < 0.001) indicated an even stronger positive association with said willingness, the quadratic term implies that this association was not linear and decreased slightly with rising climate anxiety after increasing initially (*B* = -0.07, *95% CI* [-0.11, -0.04], *p* = < 0.001). Figure 1 shows this non-linear association compared to the single linear term from model 3. Additionally, the improved model resulted in a significant coefficient for neuroticism. An increase in this variable is associated with a 0.09-point decrease in the willingness for climate-friendly behavioral change (*95% CI* [-0.15, -0.02], *p* = .011).

## Discussion

### Discussion of results

In answer to our first research question, we found that students’ neuroticism, trait anxiety, connectedness to nature, knowledge of the consequences of climate change, perceived relevance of climate change, and belief that climate change poses a personal health risk were all significantly and positively associated with their climate anxiety.

In terms of demographics, a recent meta-analysis identified a younger age and female gender as risk factors for climate anxiety [48]. In our analysis, however, age was only significant in the personality model. On average, participants aged 36–40 reported a score that was 0.42 points lower in climate anxiety than those aged 18–20, which thus aligns with the previous research indicating that a younger age is associated with higher climate anxiety-related impairment [16–21]. Consistent with this finding and our prediction, the mean climate anxiety score of German students in the present study (2.38) is higher than the mean climate anxiety scores that were reported in the general German population (1.81 [7] and 2.0 [20, 21]). Interestingly, when looking only at the 18–29 age group, the mean climate anxiety score is similar to our results (2.4 [20]), indicating that student status may not affect climate anxiety in this younger age group. Regarding gender, the results of both regression models were also consistent with previous research [7, 17, 18], including among students [3]. These results confirm that younger age and female gender are significant risk factors for heightened climate anxiety.

Regarding personality and personality-related traits, previous research found neuroticism and trait anxiety to be risk factors for heightened climate anxiety. Our results confirm this for German students as well. However, in our first regression model, trait anxiety only had a very weak, though significant, association with climate anxiety. This finding is consistent with a French study by Parmentier et al. [27], which found that trait anxiety is associated with cognitive-emotional and functional impairment due to climate anxiety. Related to trait anxiety, a study of the general German population demonstrated a significant association between general anxiety and depression and climate anxiety [7]. In terms of personality traits, our second regression model, showed that neuroticism was the only trait significantly and positively associated with climate anxiety. This finding is consistent with the results of a Polish study [26], which identified neuroticism as a weaker yet significant risk factor for cognitive-emotional impairment due to climate anxiety, as well as with a recent meta-analysis, which also revealed a significant correlation between neuroticism and climate anxiety, while none of the other personality traits (agreeableness, conscientiousness, extraversion, and openness) were significant [48]. Considering that none of the personality traits were significant in the initial MLRA, which included both trait anxiety and the personality traits; the high correlation between neuroticism, the only relevant personality trait in predicting climate anxiety, and trait anxiety; and the fact that the subsequent model, which included only trait anxiety, explained more variance than the model that included neuroticism, it is reasonable to assume that, unless there is an explicit interest in personality traits, considering only trait anxiety as a risk factor for climate anxiety in students for research or interventions could be sufficient.

In both of our regression analyses, students’ connectedness to nature was weakly but significantly and positively associated with their climate anxiety. These results are in line with the previous research findings. Whitmarsh et al. [19] found a comparably strong, significantly positive association between connectedness to nature and climate anxiety, albeit using the Nature Relatedness Scale (NR-6) [49]. Wullenkord et al. [33] also found a significant positive correlation between connectedness to nature and climate anxiety among a German sample stratified by age, gender, and level of education. The previously mentioned meta-analysis also found this significant positive association between nature relatedness and climate anxiety [48], confirming that people who feel more connected to nature constitute are at risk for heightened climate anxiety.

In our evaluation of the climate-specific health literacy scale [9] as a predictor of climate anxiety we found that not all of its subscales were significant predictors, and that these results do not entirely align with previous research. The following subscales were weakly but positively associated with climate anxiety in both regression models: knowledge of the consequences of climate change, perceived relevance of climate change, and belief that climate change poses a personal health risk. Contrary to our findings, studies have shown that knowledge of climate change and climate anxiety are negatively correlated among the general adult population in the United States and Canada [16, 28], while a meta-analysis concluded that there is no association between climate change knowledge and climate anxiety [48]. These discrepancies could be due to the type of climate knowledge. General knowledge about climate change might not relate to climate anxiety, whereas knowledge of the negative health consequences of climate changes may. The type of target group could also account for the differences in findings. When considering only students, our results are consistent with the current state of research. There is a significant correlation between climate knowledge and climate anxiety among Mexican students [3]. Furthermore, also in line with our findings, a study of German adults revealed that a higher climate risk perception predicts stronger climate anxiety [50]. This finding was also confirmed in Kühner et al.’s meta-analysis [48], which reported that climate change risk perception is one of the most frequently studied associated variables and has the strongest significant positive association with climate anxiety. Notably, however, our results show that only the perception of a personal health risk, rather than the perception of a global health risk, was a relevant predictor of climate anxiety. This suggests a discounting effect, meaning that the proximity of perceived health threats plays an important role for climate anxiety.

Since the climate-specific health literacy subscales in our study range from non-significant to significantly positive effects on climate anxiety levels, our results do not suggest that climate-specific health literacy as an overall construct protects against climate anxiety-related impairment. However, a study of nursing students in Egypt indicates that the association between a similar concept, environmental literacy, and climate anxiety is gender-specific: While environmental literacy alone had no significant effect, the significant interaction between environmental literacy and gender indicated that higher environmental literacy in women was associated with lower climate anxiety scores [51].

In answer to our second research question, we found that students’ climate anxiety, openness, and the belief that one’s own behavior impacts climate change were significantly and positively associated with their willingness for climate-friendly behavioral change. The association of climate anxiety with the willingness to engage in climate-friendly behavioral change is consistent with another German study by Wagner and Witthöft [30] that was conducted among young adults (≥16 years). In that study, climate anxiety associated with cognitive-emotional and functional impairment, was also identified as the strongest predictor of climate-friendly behavior. Although the results are not fully comparable because willingness does not always align with actual behavior [36], they are consistent.

The direction and type of relationship between climate anxiety and pro-environmental behavior is controversial, however. For example, a German study by Wullenkord et al. [7] identified pro-environmental intentions as the strongest predictor of climate anxiety, while another German study found that climate anxiety and pro-environmental behavior precede each other in time and positively predict each other [43]. In addition, previous studies have examined whether there is an inverted U-shaped relationship between climate anxiety and pro-environmental behavior. This relationship is based on the following heuristic: motivation for pro-environmental behavior is weak when climate anxiety is very low because people do not see an urgent need to act. Conversely, when climate anxiety is very high, it can lead to psychological impairments and hinder the ability to engage in pro-environmental behavior. In the middle of the curve lies the so-called ‘Goldilocks zone of climate anxiety’, where climate anxiety is sufficient to motivate pro-environmental behavior without limiting the ability to act [52]. While two Australian studies support this hypothesis [53, 54], a German study rejects it [30]. The present study found only a slight inverted U-shaped relationship between climate change and the willingness for climate-friendly behavioral change among German students of health-related subjects when a quadratic term for climate anxiety was added to the analysis. As mentioned above, it must be taken into account that willingness does not always correspond to actual behavior [36], so this relationship could only be tested to a limited extent in this study. Nevertheless, Maduneme [55] also found an inverted U-shaped relationship between climate anxiety and pro-environmental behavioral intentions among students in the United States.

Previous research has associated all five of the BFI-10 personality traits with planned, intended, or actual climate-friendly or pro-environmental behavior, though never all at once [25, 26, 37, 38]. Our results, however, indicate a significant relationship only between students’ openness and their willingness for climate-friendly behavioral change. This may be due to the differences between actual behavior, planned or intended behavior, and willingness for behavioral change. The willingness to change behavior may depend solely on openness, whereas the actual planning and execution of behavior may also depend on other personality traits. Nevertheless, our results are somewhat in line with the results of Tucholska et al. [26], who identified a significant positive association between openness and planned pro-environmental behavior in Polish adults. This association is stronger than the association between openness and willingness for climate-friendly behavioral change in the present study. While Tucholska et al. [26] used a comparable instrument to assess personality traits [56], they developed their own instrument to operationalize planned pro-environmental behavior. Thus the comparability of the results is limited. As mentioned, other studies have demonstrated a positive correlation between openness and actual pro-environmental behavior [37, 38]. Overall, openness appears to be a personality trait that promotes the willingness to change behavior and also to plan and execute this change.

We found that believing one’s own behavior has an impact is strongly associated with the willingness for climate-friendly behavioral change. This is similar to what previous research indicates. For example, a study involving Italian adults found that climate anxiety has both a positive direct effect on pro-environmental behavior and a negative indirect effect on pro-environmental behavior, which is mediated by impairments in self-efficacy [57]. Also consistent with our findings, a study of German university students revealed that direct goal self-efficacy (e.g., “As an individual, I can promote environmental protection”) significantly and positively predicts the intention to engage in private pro-environmental behaviors (e.g., “In the future, I intend to use recycled paper if possible”) [58]. Thus, efficacy beliefs seem to be a viable factor concerning environmental behavior in the context of climate change and associated anxieties, and could be considered for use in intervention design.

### Strengths and limitations

This study addresses a notable research gap by investigating risk and protective factors for functional impairment due to climate anxiety among German students of health-related subjects, as well as factors associated with these student’s willingness to adopt climate-friendly behavioral changes.

However, the study has some limitations. Due to its cross-sectional design, it is not possible to infer any causal relationships. Additionally, bias resulting from self-reported data and a potential overrepresentation of students particularly interested in climate change cannot be ruled out. Nevertheless, the climate anxiety score obtained in this study is comparable to that in an earlier study by Hajek and König [20].

The BFI-10 [46] used in this study showed mixed internal consistency, despite being considered an established assessment tool. Additionally, the climate-specific health literacy indicators (knowledge of climate change consequences, relevance of climate change, action efficacy, climate change as a global risk, and climate change as a personal risk) are based on single items, which could limit their validity. When using the CAS [7] to measure climate anxiety, it should be noted that it is based on a rather pathological understanding. According to Wullenkord et al. [7], the scale primarily measures climate-related emotional impairments. However, the authors also recommend placing greater emphasis on milder forms of climate anxiety. Clinical cut-off scores have already been established for the English version of the CAS [59]. However, as no cut-off scores have been established yet for the German version of the CAS, we did not assess the clinical relevance of the climate anxiety scores in this study.

Chan et al. [60] demonstrated in a longitudinal study that negative emotions toward climate change can lead to cognitive-emotional and functional impairments. Therefore, early identification and targeted interventions for milder forms of climate anxiety could prevent a future worsening of symptoms. In light of the methodological limitations outlined above, it is advisable to use more differentiated instruments to measure climate anxiety in future studies. The newly developed ICE [61] comprises eight different emotional response to climate change, offering a promising approach for this purpose. Furthermore, longitudinal studies examining the development of climate-specific health literacy in relation to climate anxiety throughout the course of studies are recommended.

We also collected data from people who identified as diverse. Due to the small sample size (*n* = 10), however, we cannot make any valid statements in this regard. Nevertheless, our data suggest that this group has the highest average level of climate anxiety (female = 2.57, male = 1.93, diverse = 2.81), though the difference is not significant.

Although we asked about the willingness for climate-friendly behavioral change, we did not inquire about their prior climate-friendly actions. Consequently, the results are somewhat limited as individuals who are already exhibiting very climate-friendly behavior have limited options for change. Lastly, it should be noted that we did not pre-register our study before its realization, which limits its replicability to some extent. However, the study data is available online, and we will share the analysis syntax upon request.

### Recommendations for action

One of the aims of our study was to identify risk and protective factors for emotional impairment due to climate anxiety in students, particularly among students of health-related subjects. To this end, we tested a questionnaire on climate-specific health literacy [9] as a potential protective factor. As the climate-specific health literacy subscales had either a significant positive effect or no significant effect on the level of climate anxiety, our results do not suggest that climate-specific health literacy protects against climate anxiety impairment overall. Other studies suggest that gender should also be considered when analyzing this relationship [51], which our results confirm.

The significant positive association between perceived knowledge of the consequences of climate change and the fact that students of health-related subjects learn about the health consequences of climate change as part of their studies underscores this target groups’ potential vulnerability to climate-related stressors. Therefore, future research should continue identifying protective factors to develop a basis for intervention development to protect against impairment because of climate anxiety. Interventions should equip students of health-related subjects, whose courses include information about the health consequences of climate change, with the skills to appropriately process and utilize climate emotions for the purpose of climate action. Since our results show that young age, female gender, connectedness to nature, trait anxiety, and neuroticism are possible risk factors for climate anxiety impairment, students of health-related subjects with these characteristics in particular should be surveyed or involved in a participatory manner when developing interventions to best reach them. Nevertheless, it should not be disregarded that a certain level of concern about climate change is only reasonable, not pathological, and that there is a fundamental need for appropriate and effective societal climate protection measures [4].

As our study is limited to students of health-related subjects, future research could investigate whether students of different subjects (e.g., health- or environmental-related vs. non-health- or environmental-related) differ in terms of their climate anxiety and their climate-specific health literacy. Longitudinal studies could also track students’ climate anxiety alongside their knowledge development throughout their study progression. However, it should be noted that we did not find higher levels of climate anxiety among our sample than among a general sample of the same age group in another study. Therefore, future research comparing students and non-student within the same age group in a single study could be worthwhile.

Another aim of the study was to examine the association between students’ willingness to make climate-friendly behavioral changes in order to identify potential starting points for promoting sustainable behavior. Since our results revealed a significant association between openness and behavioral change, interventions aimed at fostering climate-friendly actions could be specifically target students exhibiting this trait. When developing and evaluating appropriate interventions, the personality trait of openness should be considered. In addition, ways to motivate less open-minded students to engage in climate-friendly behavior should be investigated. These interventions could promote of the belief that one’s own behavior has an impact on climate change, as our results indicated that this belief is significantly associated with students’ willingness for climate-friendly behavioral change. Given that our results also indicate a strong and significant association between climate anxiety and climate-friendly behavioral change (see Table 3), and considering the controversial nature and direction of this relationship (see Sect.  5.1), future research should explore this connection further to clarify the extent to which climate anxiety either motivates or hinders climate action.

## Conclusion

Our study identified potential risk factors for climate anxiety impairment in students, including young age, female gender, anxiety, neuroticism, and connectedness to nature. However, we could not identify climate-specific health literacy as a potential protective factor. In fact, we found the opposite to be true. Factors that may promote climate-friendly behavioral changes among students include climate anxiety, openness, and the perceived action efficacy. Concerning willingness to change behavior, we found that adding a quadratic term for climate anxiety indicates a non-linear relationship between climate anxiety and willingness. Initially, willingness increases with rising climate anxiety, but then it decreases slightly. Further exploration of the nature and direction of the relationship between climate anxiety and climate-friendly behavior is necessary to more precisely determine the extent to which climate anxiety motivates or paralyzes. In view of advancing climate change and the increasing importance of its health consequences in the course content of students of health-related subjects, further research on protective factors regarding climate anxiety is also necessary, both to safeguard students’ mental health and to ensure that climate action is not impeded. The need for comprehensive societal strategies to mitigate and adapt to climate change is not undermined by these considerations but rather emphasized. However, our findings should be interpreted with some caution. The indicators of climate-specific health literacy were collected using single items, and the personality questionnaire showed mixed internal consistency.

## Supplementary Information

Supplementary Material 1.

## Data Availability

The datasets generated and analyzed during the current study are included in this published article supplementary material (Additional file 1.sav).

## Appendix

Power calculation (G*Power 3.1.9.7):

F tests - Linear multiple regression:

- Fixed model, R² deviation from zero

Analysis:

- A priori: Compute required sample size

Input:

- Effect size f² = 0.085
- α err prob = 0.05
- Power (1-β err prob) = 0.95
- Number of predictors = 29

Output:

- Noncentrality parameter λ = 36.9750000
- Critical F = 1.4956047
- Numerator df = 29
- Denominator df = 405
- Total sample size = 435

## Abbreviations

BFI-10: Big Five Inventory
CAS: Climate Anxiety Scale
CNS-A: Connectedness to Nature Scale for Adolescents
ICE: Inventory of Climate Emotions
MLRA: Multiple Linear Regression Analyses
NR-6: Nature Relatedness Scale
STAI: State-Trait-Anxiety Inventory

## References

[1] Ogunbode CA, Doran R, Hanss D, Ojala M, Salmela-Aro K, Van Den Broek KL, et al. Climate anxiety, wellbeing and pro-environmental action: correlates of negative emotional responses to climate change in 32 countries. J Environ Psychol. 2022;84:101887. 10.1016/j.jenvp.2022.101887.

[2] Hickman C, Marks E, Pihkala P, Clayton S, Lewandowski RE, Mayall EE, et al. Climate anxiety in children and young people and their beliefs about government responses to climate change: a global survey. Lancet Planet Health. 2021;5(12):e863–73. 10.1016/S2542-5196(21)00278-3. 34895496 10.1016/S2542-5196(21)00278-3

[3] Ramírez-López AS, Rosetti MF, Poma A. Gender, exposure to news, knowledge about climate change, and prosociality predict climate anxiety scores in Mexican students. Ecopsychology. 2023;15(2):184–92. 10.1089/eco.2022.0049.

[4] Clayton S. Climate anxiety: psychological responses to climate change. J Anxiety Disord. 2020;74:102263. 10.1016/j.janxdis.2020.102263. 32623280 10.1016/j.janxdis.2020.102263

[5] Heeren A, Asmundson GJG. Understanding climate anxiety: what decision-makers, health care providers, and the mental health community need to know to promote adaptative coping. J Anxiety Disord. 2023;93:102654. 10.1016/j.janxdis.2022.102654. 36414530 10.1016/j.janxdis.2022.102654

[6] Clayton S, Karazsia BT. Development and validation of a measure of climate change anxiety. J Environ Psychol. 2020;69:101434. 10.1016/j.jenvp.2020.101434.

[7] Wullenkord MC, Tröger J, Hamann KRS, Loy LS, Reese G. Anxiety and climate change: a validation of the Climate Anxiety Scale in a German-speaking quota sample and an investigation of psychological correlates. Clim Change. 2021;168:1–23. 10.1007/s10584-021-03234-6.

[8] Reismann L, Weber A, Leitzmann M, Jochem C. Climate-specific health literacy and medical advice: The potential for health co-benefits and climate change mitigation. An exploratory study. J Clim Change Health. 2021;4:100072. 10.1016/j.joclim.2021.100072.

[9] Albrecht L, Reismann L, Leitzmann M, Bernardi C, Von Sommoggy J, Weber A, et al. Climate-specific health literacy in health professionals: an exploratory study. Front Med. 2023;10:1236319. 10.3389/fmed.2023.1236319. 10.3389/fmed.2023.1236319 PMC10622978 37928468

[10] Pihkala P. Anxiety and the ecological crisis: an analysis of eco-anxiety and climate anxiety. Sustainability. 2020;12(19):7836. 10.3390/su12197836.

[11] Tam KP, Chan HW, Clayton S. Climate change anxiety in China, India, Japan, and the United States. J Environ Psychol. 2023;87:101991. 10.1016/j.jenvp.2023.101991.

[12] Gago T, Sargisson RJ, Milfont TL. A meta-analysis on the relationship between climate anxiety and wellbeing. J Environ Psychol. 2024;94:102230. 10.1016/j.jenvp.2024.102230.

[13] Söder A, Herr RM, Görig T, Diehl K. Climate change worry in German university students: Determinants and associations with health-related outcomes. Climate. 2025;13(2):27. 10.3390/cli13020027.

[14] Schwaab L, Gebhardt N, Friederich HC, Nikendei C. Climate change related depression, anxiety and stress symptoms perceived by medical students. Int J Environ Res Public Health. 2022;19(15):9142. 10.3390/ijerph19159142. 35897512 10.3390/ijerph19159142 PMC9332784

[15] Marczak M, Wierzba M, Zaremba D, Kulesza M, Szczypiński J, Kossowski B, et al. Beyond climate anxiety: development and validation of the inventory of climate emotions (ICE): a measure of multiple emotions experienced in relation to climate change. Glob Environ Change. 2023;83:102764. 10.1016/j.gloenvcha.2023.102764.

[16] Asgarizadeh Z, Gifford R, Colborne L. Predicting climate change anxiety. J Environ Psychol. 2023;90:102087. 10.1016/j.jenvp.2023.102087.

[17] Heeren A, Mouguiama-Daouda C, Contreras A. On climate anxiety and the threat it may pose to daily life functioning and adaptation: a study among European and African French-speaking participants. Clim Change. 2022;173(1):15. 10.1007/s10584-022-03402-2. 35912274 10.1007/s10584-022-03402-2 PMC9326410

[18] Sangervo J, Jylhä KM, Pihkala P. Climate anxiety: conceptual considerations, and connections with climate hope and action. Glob Environ Change. 2022;76:102569. 10.1016/j.gloenvcha.2022.102569.

[19] Whitmarsh L, Player L, Jiongco A, James M, Williams M, Marks E, et al. Climate anxiety: what predicts it and how is it related to climate action? J Environ Psychol. 2022;83:101866. 10.1016/j.jenvp.2022.101866.

[20] Hajek A, König HH. Climate anxiety in Germany. Public Health. 2022;212:89–94. 10.1016/j.puhe.2022.09.007. 36272204 10.1016/j.puhe.2022.09.007

[21] Hajek A, König HH. Climate anxiety and mental health in Germany. Climate. 2023;11(8):158. 10.3390/cli11080158.

[22] Gago T, Sá I. Environmental worry and wellbeing in young adult university students. Curr Res Environ Sustain. 2021;3:100064. 10.1016/j.crsust.2021.100064.

[23] Selçuk Tosun A, Ünsal Yüceer Ü, Demirdağ B, Akgül Gündoğdu N, Lök N. Climate change worry and environmental sensitivity among nursing students. Public Health Nurs. 2025;42(5):1661–8. 10.1111/phn.13580. 40512470 10.1111/phn.13580

[24] Daeninck C, Kioupi V, Vercammen A. Climate anxiety, coping strategies and planning for the future in environmental degree students in the UK. Front Psychol. 2023;14:1126031. 10.3389/fpsyg.2023.1126031. 37564302 10.3389/fpsyg.2023.1126031 PMC10409990

[25] Cipriani E, Frumento S, Gemignani A, Menicucci D. Personality traits and climate change denial, concern, and proactivity: A systematic review and meta-analysis. J Environ Psychol. 2024;95:102277. 10.1016/j.jenvp.2024.102277.

[26] Tucholska K, Gulla B, Ziernicka-Wojtaszek A. Climate change beliefs, emotions and pro-environmental behaviors among adults: the role of core personality traits and the time perspective. PLoS ONE. 2024;19(4):e0300246. 10.1371/journal.pone.0300246. 38598437 10.1371/journal.pone.0300246 PMC11006203

[27] Parmentier ML, Weiss K, Aroua A, Betry C, Rivière M, Navarro O. The influence of environmental crisis perception and trait anxiety on the level of eco-worry and climate anxiety. J Anxiety Disord. 2024;101:102799. 10.1016/j.janxdis.2023.102799. 38091939 10.1016/j.janxdis.2023.102799

[28] Thomson EE, Roach SP. The relationships among nature connectedness, climate anxiety, climate action, climate knowledge, and mental health. Front Psychol. 2023;14:1–11. 10.3389/fpsyg.2023.1241400. 10.3389/fpsyg.2023.1241400 PMC10684686 38034293

[29] Schwartz SEO, Benoit L, Clayton S, Parnes MF, Swenson L, Lowe SR. Climate change anxiety and mental health: environmental activism as buffer. Curr Psychol. 2023;42(20):16708–21. 10.1007/s12144-022-02735-6. 10.1007/s12144-022-02735-6 PMC8883014 35250241

[30] Wagner L, Witthöft M. Klimaangst – angebracht oder dysfunktional? eine Untersuchung des Zusammenhangs mit Depressivität, Ängstlichkeit und positiven klimabezogenen Verhaltensweisen. Z Für Klin Psychol Psychother. 2024;53(2):59–71. 10.1026/1616-3443/a000754.

[31] Düllberg G, Rinderhagen M, Hennig-Fast K, Mc Call T. Climate anxiety in adults with depression and/or anxiety disorders: a German quantitative study. BMC Psychol. under review.

[32] Curll SL, Stanley SK, Brown PM, O’Brien LV. Nature connectedness in the climate change context: Implications for climate action and mental health. Transl Issues Psychol Sci. 2022;8(4):448–60.

[33] Wullenkord MC, Johansson M, Loy LS, Menzel C, Reese G. Go out or stress out? Exploring nature connectedness and cumulative stressors as resilience and vulnerability factors in different manifestations of climate anxiety. J Environ Psychol. 2024;95:102278. 10.1016/j.jenvp.2024.102278.

[34] Fang WT, Hassan A, LePage BA. Environmental Literacy. In: Fang WT, Hassan A, LePage BA, editors. The Living Environmental Education. Singapore: Springer Nature Singapore; 2023. pp. 93–126.

[35] Çolak M, Dogan R, Dogan S. Effect of climate change and health course on global warming knowledge and attitudes, environmental literacy, and eco-anxiety level of nursing students: A quasi‐experimental study. Public Health Nurs. 2025;42:1315–24. 10.1111/phn.13536. 39822057 10.1111/phn.13536 PMC12001001

[36] Meyer F, Shamon H, Vögele S. Dynamics and heterogeneity of environmental attitude, willingness and behavior in Germany from 1993 to 2021. Sustainability. 2022;14(23):16207. 10.3390/su142316207.

[37] Freminot K, Major-Smith K, Northstone K, Halstead I, Major-Smith D. Associations between Big-5 personality traits, cognitive ability, and climate beliefs and behaviours: Results from a longitudinal UK birth cohort. Eur J Psychol. 2024;20(4):288–302. 10.5964/ejop.13657. 39678302 10.5964/ejop.13657 PMC11636718

[38] Soutter ARB, Bates TC, Mõttus R. Big Five and HEXACO personality traits, proenvironmental attitudes, and behaviors: A meta-analysis. Perspect Psychol Sci. 2020;15(4):913–41. 10.1177/1745691620903019. 32384257 10.1177/1745691620903019 PMC7333518

[39] Kurth C, Pihkala P. Eco-anxiety: what it is and why it matters. Front Psychol. 2022;13:981814. 10.3389/fpsyg.2022.981814. 36211934 10.3389/fpsyg.2022.981814 PMC9537110

[40] Bhullar N, Davis M, Kumar R, Nunn P, Rickwood D. Climate anxiety does not need a diagnosis of a mental health disorder. Lancet Planet Health. 2022;6(5):e383. 10.1016/S2542-5196(22)00072-9. 35550075 10.1016/S2542-5196(22)00072-9

[41] Hogg TL, Stanley SK, O’Brien LV, Wilson MS, Watsford CR. The Hogg Eco-Anxiety Scale: Development and validation of a multidimensional scale. Glob Environ Change. 2021;71:102391. 10.1016/j.gloenvcha.2021.102391.

[42] Karl JA, Stanley SK. Is mindfulness a double-edged sword? Associations with climate anxiety and pro-environmental behavior. Mindfulness. 2024;15(9):2207–17. 10.1007/s12671-024-02427-1.

[43] Kühner C, Rudolph CW, Zacher H. Reciprocal relations between climate change anxiety and pro-environmental behavior. Environ Behav. 2024;56(5–6):408–39. 10.1177/00139165241297050.

[44] Weber A, Kroiss K, Reismann L, Jansen P, Hirschfelder G, Sedlmeier AM, et al. Health-promoting and sustainable behavior in university students in Germany: A cross-sectional study. Int J Environ Res Public Health. 2023;20(7):5238. 10.3390/ijerph20075238. 37047853 10.3390/ijerph20075238 PMC10094390

[45] Grimm J. State-Trait-Anxiety Inventory nach Spielberger. Deutsche Lang- und Kurzversion. Methodenforum der Universität Wien: MF-Working Paper 2009/02; 2009.

[46] Rammstedt B, John OP. Measuring personality in one minute or less: A 10-item short version of the Big Five Inventory in English and German. J Res Personal. 2007;41(1):203–12. 10.1016/j.jrp.2006.02.001.

[47] Götting K, Böhme T, Geiger SM. Connectedness to Nature Scale – Adolescents (CNS-A) Entwicklung und Validierung einer Skala zur Erfassung von Naturverbundenheit bei Jugendlichen. Umweltpsychologie. 2019;23(2):131–50.

[48] Kühner C, Gemmecke C, Hüffmeier J, Zacher H. Climate change anxiety: A meta-analysis. Glob Environ Change. 2025;93:103015. 10.1016/j.gloenvcha.2025.103015.

[49] Nisbet EK, Zelenski JM. The NR-6: a new brief measure of nature relatedness. Front Psychol. 2013;4. 10.3389/fpsyg.2013.00813. 10.3389/fpsyg.2013.00813 PMC3814587 24198806

[50] Reese G, Rueff M, Wullenkord MC. No risk, no fun… ctioning? Perceived climate risks, but not nature connectedness or self-efficacy predict climate anxiety. Front Clim. 2023;5:1158451. 10.3389/fclim.2023.1158451.

[51] Amin SM, El-Monshed AH, Khedr MA, Morsy OMI, El‐Ashry AM. Future nurses in a changing climate: Exploring the relationship between environmental literacy and climate anxiety. J Adv Nurs. 2024;16606. 10.1111/jan.16606. 10.1111/jan.16606 39524017

[52] Heeren A, Clayton S. Searching for the Goldilocks zone of climate anxiety. Trends Cogn Sci. 2026;30(1):1–3. 10.1016/j.tics.2025.11.007. 41436321 10.1016/j.tics.2025.11.007

[53] Coates Z, Brown S, Kelly M. Understanding climate anxiety and potential impacts on pro-environment behaviours. J Anxiety Disord. 2025;114:103049. 10.1016/j.janxdis.2025.103049. 40540828 10.1016/j.janxdis.2025.103049

[54] Hogg TL, Stanley SK, O’Brien LV, Watsford CR, Walker I. Clarifying the nature of the association between eco-anxiety, wellbeing and pro-environmental behaviour. J Environ Psychol. 2024;95:102249. 10.1016/j.jenvp.2024.102249.

[55] Maduneme E. Some slice of climate anxiety … is good: a cross-sectional survey exploring the relationship between college students media exposure and perceptions about climate change. J Health Commun. 2024;29(sup1):45–56. 10.1080/10810730.2024.2354370. 38775847 10.1080/10810730.2024.2354370

[56] Schupp J, Gerlitz JY. Big Five Inventory-SOEP (BFI-S). Zusammenstellung Sozialwissenschaftlicher Items Skalen ZIS. 2008. 10.6102/ZIS54.

[57] Innocenti M, Santarelli G, Lombardi GS, Ciabini L, Zjalic D, Di Russo M, et al. How can climate change anxiety induce both pro-environmental behaviours and eco-paralysis? The mediating role of general self-efficacy. Int J Environ Res Public Health. 2023;20(4):3085. 10.3390/ijerph20043085. 36833780 10.3390/ijerph20043085 PMC9960236

[58] Hamann KRS, Reese G. My influence on the world (of others): Goal efficacy beliefs and efficacy affect predict private, public, and activist pro-environmental behavior. J Soc Issues. 2020;76(1):35–53. 10.1111/josi.12369.

[59] Cosh SM, Williams SE, Lykins AD, Bartik W, Tully PJ. Detecting and classifying eco-anxiety: development of clinical cut-off scores for the climate change anxiety scale. BMC Psychol. 2024;12(1):738. 10.1186/s40359-024-02240-4. 39696553 10.1186/s40359-024-02240-4 PMC11657576

[60] Chan HW, Lin L, Tam KP, Hong Y. yi. From negative feelings to impairments: A longitudinal study on the development of climate change anxiety. J Anxiety Disord. 2024;107:102917. 10.1016/j.janxdis.2024.102917 10.1016/j.janxdis.2024.102917 39217778

[61] Marczak M, Wierzba M, Kossowski B, Marchewka A, Morote R, Klöckner CA. Emotional responses to climate change in Norway and Ireland: a validation of the Inventory of Climate Emotions (ICE) in two European countries and an inspection of its nomological span. Front Psychol. 2024;15:1211272. 10.3389/fpsyg.2024.1211272. 38390416 10.3389/fpsyg.2024.1211272 PMC10881694

